# The role of Bach2 in regulating CD8 + T cell development and function

**DOI:** 10.1186/s12964-024-01551-8

**Published:** 2024-03-08

**Authors:** Xinyu Weng, Min Zheng, Yanning Liu, Guohua Lou

**Affiliations:** The State Key Laboratory for Diagnosis and Treatment of Infectious Diseases, National Clinical Research Center for Infectious Diseases, Collaborative Innovation Center for Diagnosis and Treatment of Infectious Diseases, The First Affiliated Hospital, College of Medicine, Zhejiang University, 79# Qingchun Road, 6A-5, Hangzhou, 310003 China

**Keywords:** Bach2, CD8, Lymphocytes, Immune diseases

## Abstract

Bach2 was initially discovered in B cells, where it was revealed to control the transcription involved in cell differentiation. Bach2 is intimately connected to CD8 + T lymphocytes in various differentiation states and subsets according to recent findings. Bach2 can regulate primitive T cells, stimulate the development and differentiation of memory CD8 + T cells, inhibit the differentiation of effector CD8 + T cells, and play a significant role in the exhaustion of CD8 + T cells. The appearance and development of diseases are tightly linked to irregular CD8 + T cell differentiation and function. Accordingly, Bach2 offers novel approaches and possible targets for the clinical treatment of associated disorders based on research on these pathways. Here, we summarize the role of Bach2 in the function and differentiation of CD8 + T cells and its potential clinical applications.

## Background

Bach2 is a highly conserved member of the basic and leucine zipper domain (bZIP) superfamily of transcription factors (TFs); it regulates transcriptional activity in T- and B-lymphocyte differentiation and maturation at super enhancers or regions of high transcriptional activity. To better elucidate the biological function of Bach2 in clinical pathology, it is important to clarify the precise regulatory mechanisms of Bach2 transcriptional and post-transcriptional modulation [1].

Orchestration of B cell differentiation by Bach2 has been widely demonstrated. Bach2 is a crucial tissue factor that regulates immunity [2]. The activated B cell immunoglobulin gene undergoes class switching recombination (CSR) and somatic hypermutation (SHM), which are both critical processes involving Bach2. Numerous studies have also demonstrated that Bach2 is required for CD4 + T cell development and maturation.

Long-lasting protective immunity can be provided by CD8 + T cells, which are essential for preventing intracellular infections and limiting the proliferation of malignant cells. The metabolism of CD8 + T cells during infection involves numerous transcriptional, translational, and epigenetic alterations. CD8 + T cell-mediated immunity is impacted by any alteration in overall metabolism [3]. Furthermore, a number of recent studies have gradually reported the crucial function of Bach2 as a regulator of CD8 + T cells. An increasing number of discoveries are being made, particularly regarding the associations of various diseases, such as cancer and immune system diseases. Thus, it is crucial to fully characterize the regulatory impact of Bach2 on CD8 + T cells.

In this review, we discuss the role of Bach2 in orchestrating the differentiation and function of effector and memory CD8 + T cells, as well as the exhaustion state of T cells and related diseases.

### Biological structure and function of Bach2

Bach2 is a basic region-leucine zipper (bZip) transcription factor that forms heterodimers with small Maf oncoproteins and binds to target genes, thus repressing their expression [4]. Bach2 is located on human chromosome 6 (6q15) and mouse chromosome 4 [5].

Bach2 function has been most extensively investigated in B cells. It is known to repress the expression of B lymphocyte–induced maturation protein 1 (Blimp-1), also known as PR domain zinc finger protein 1 (PRDM1), by binding to the Maf recognition element (MARE) 59 of the PRDM1 (Blimp-1) gene [6]. MARE contains a binding motif for activator protein-1 (AP-1) factors (dimers of Fos and Jun proteins), thus suggesting that Bach2 affects AP-1-mediated gene regulation and vice versa [5]. AP-1 family transcription factors (TFs) play a central role in transducing T-cell receptor (TCR)-driven effector programs [7]. The availability of AP-1 sites to Jun TFs allows TCR-driven effector programs to be modulated in a stage-specific and contextual manner in CD8 + T cells, allowing for the generation of transcriptionally intermediate memory cells [7]. Bach2 inhibits interferon regulatory Factor 4 (IRF4)-dependent transcription by competing with the DNA-binding activity of IRF4 and restricting chromatin accessibility, which helps to maintain regulatory T-cell (Treg) homeostasis and balance TCR signalling [8]. Bach2 increases the generation and functionality of Tregs to balance immunological activity in the T helper 2 (Th2) cell immune response and associated inflammatory disorders by preventing the development of CD4 T cells into Th2 cells [9]. Several studies have shown that Bach2 is a vitamin D-induced protein that regulates a significant portion of the vitamin D transcriptome; more importantly, it plays a key role in stimulating the expression of interleukin 10 (IL-10) [10]. Overall, Bach2 plays a biological role in immunometabolism as a transcription suppressor that influences the process of genetic transcription through several signalling pathways.

### The role of Bach2 in regulating CD8 + T cell development and function

More information on how cytokine signalling and other external factors affect Bach2 activity and function in regulatory T cells will be provided below. The following sections will also detail the precise connections and regulatory processes involved in the interaction between Bach2 and CD8 + T cells.

#### Bach2-mediated regulation of naive T cells

Naive T cells, progenitors of effector T cells and memory T cells, have a low metabolic rate and inability to trigger effector immune responses. After birth, naive T cells predominate, but their proportion decreases after adolescence [11]. 

Bach2 deficiency results in a reduction in naive T cells and enhances effector-memory T cells. Thus, Bach2 suppresses effector memory-related genes, such as C-C motif chemokine receptor 4 (CCR4), growth stimulation expressed gene 2 (ST-2), S100 calcium binding protein a (S100a) and Blimp-1, to maintain the naive T-cell state and regulate the generation of effector-memory T cells. Bach2 is upregulated during differentiation and functions to regulate homeostasis of peripheral T cells by suppressing the expression of effector memory-related genes in naive T cells [8]. Moreover, Bach2 can maintain the naive state of T cells and maintain the homeostasis of peripheral T cells by inhibiting the expression of effector memory-related genes in naive T cells [12].

The regulatory effect of Bach2 on naive T cells is mainly reflected in the differentiation of CD8 + T cells. Bach2 engages in passive transcriptional repression through competition with AP-1 factors for DNA binding. Bach2 regulates CD8 + T cell differentiation by controlling the access of AP-1 factors to enhancers The regulation of CD8 + T cells by Bach2 in response to viral infection lays the foundation for further studies on how Bach2 controls CD8 + T cell differentiation and function [7].

Overall, Bach2 regulates the development of effector memory T cells by suppressing genes involved in effector memory to maintain the condition of naive T cells. These results establish the groundwork for further research on the function of Bach2 in controlling CD8 + T cell development and function.

#### Bach2-mediated regulation of CD8 + T cells

Bach2 expression on T cells is necessary for the development of T cell regulatory functions and subsequent control; furthermore, it affects how peripherally generated regulatory T cells are handled. A progressive wasting condition known as experimental Bach2 knockout is characterized by an increase in antinuclear antibodies and a substantial decrease in the number of regulatory T cells [13]. In Bach2-deficient animals, regulatory T cells exhibited a terminally differentiated phenotype after being partially restored to the normal range. Bach2 loss drastically reduces peripherally triggered regulatory T cell development, which results in the activation of T cells by several genes linked to the atypical T cell subgroup [14].

Intriguingly, Bach2 may play various roles based on the maturation checkpoint or T-cell growth phase [15]. As a part of the process of T cell development and proliferation, naive T cells can proliferate and develop into various types of effector and memory T cells in response to their interactions with cognate antigens in the periphery. These cells can then move to different tissues for local antigen surveillance [16]. Based on their homing and functional characteristics, memory T cells are further divided into two groups: central memory T cells and effector memory T cells [12]. Bach2 has a variety of functions in T cells, but the reasons behind these functions have yet to be determined. It is also unknown whether the functional changes in Bach2 are specifically related to the stage at which T cells proliferate.

To better elucidate the biological function of Bach2 in clinical pathology, it is important to clarify the precise regulatory mechanisms of Bach2 transcriptional and post-transcriptional modulation. Menin functions as an epigenetic regulator to maintain histone acetylation on the Bach2 gene, which increases Bach2 expression in T cells and suppresses the development of the potentially harmful secretory phenotype associated with ageing in T cells [1].

The thymus and peripheral T cells are often where Bach2 mRNA is found. The transcriptional increase in Bach2 also steadily increased as T cells differentiated in the thymus. The amount of Bach2 transcripts in antigen-presenting T cells is much lower than that in other T cells according to experimental studies, although even the regulatory factors governing Bach2 once fully matured T cells have not yet been identified. Even when the spliceosome sequence was similar, Bach2 expression in T cells was relatively lower than that in B cells [12].

**Fig. 1:**
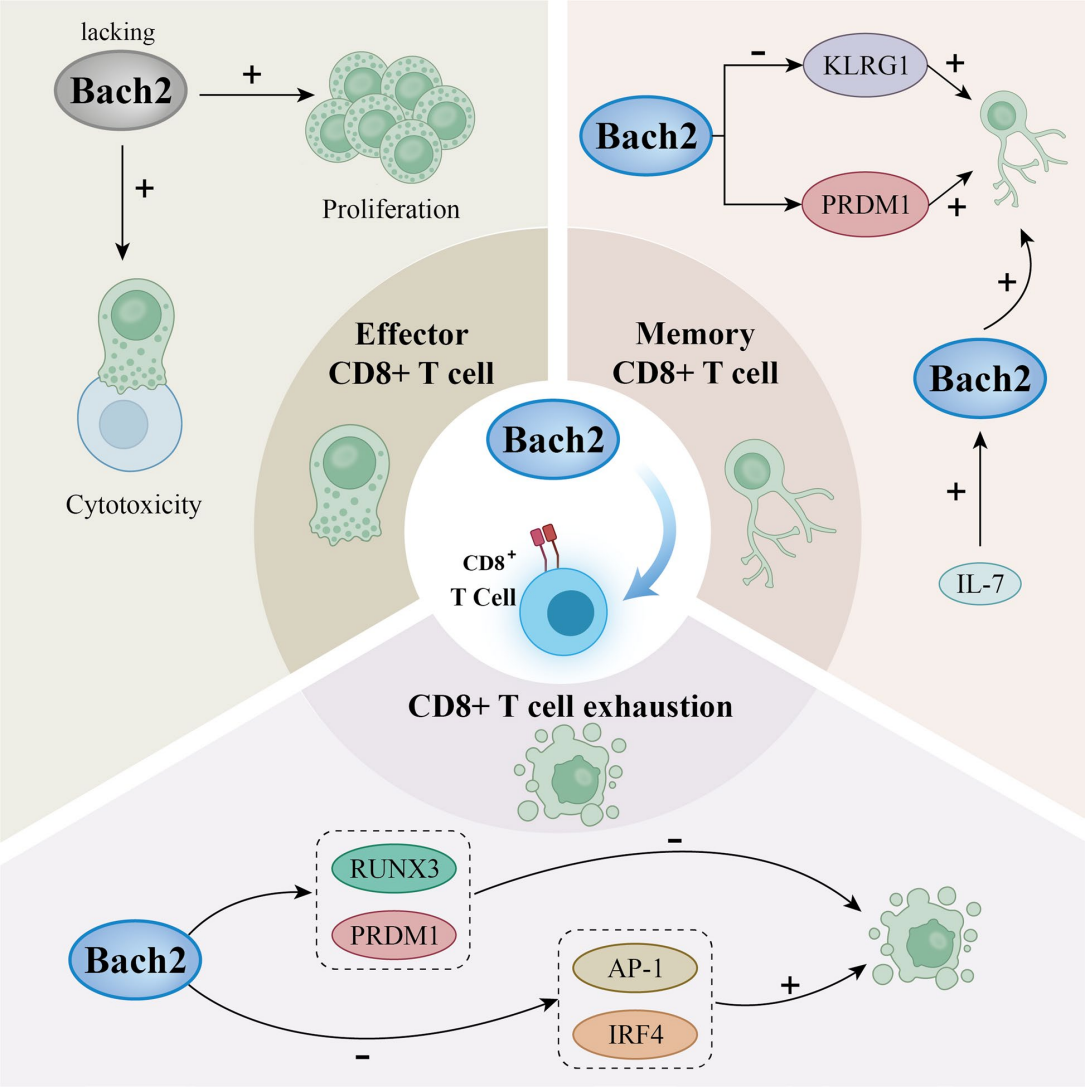
Effect of Bach2 on different types of CD8+ T cells. In the absence of Bach2, murine effector CTLs activated in vitro exhibited enhanced proliferative and killing capacities. Through PRDM1, Bach2 encourages the growth and recall proliferation of memory CD8+ T cells. IL-7 increased the expression of Bach2, a key transcription factor produced by long-lived memory T cells. Bach2 promotes the proliferation of memory CD8+ T cells through PRDM1 and KLRG1. Bach2 may influence CD8+ T cell development by affecting PRDM1 expression and the RUNX3 pathway, which prevents terminal exhaustion. Bach2 promotes exhaustion by limiting the function of IRF4 and the AP-1 family of transcription factors when it is overexpressed

More information on how cytokine signalling and other external factors affect Bach2 activity and function in regulatory T cells will be provided below. The following sections detail the precise connections and regulatory processes involved in the interaction between Bach2 and CD8 + T cells (Fig. 1).

##### Bach2-mediated regulation of effector CD8 + T cells

Different functional T cell subsets manage multiple facets of immune defence against various bacterial infections, viral infections, and diseases of the autoimmune system. Numerous recent experimental results indicate that Bach2 is vital for regulating the differentiation of effector CD8 + T cells into cells with diverse roles. Downregulation of Bach2 in naive CD8 + T cells is essential for the complete differentiation of effector cells.

According to some reports, despite the activation of their phenotypes, T cells lacking Bach2 might not generate complete effector capability. Bach2 is thought to actively suppress T cell activation during homeostasis, cytokine-driven turnover, and maintenance. In comparison, T cells lacking Bach2 naturally exhibit an independent activation phenotype to cope with the new environment. In conclusion, T lymphocytes lacking Bach2 are unable to perform dominant roles or exhibit unique antimicrobial properties related to effectors or memory [12].

It is unclear what governs Bach2 expression to enhance the differentiation of certain T cell subsets. Similarly, the activity of adequately differentiated mature T lymphocytes is significantly impacted by Bach2.

Effector cytotoxic T lymphocytes (CTLs) activated in vitro from mice were shown to have greater proliferative and cytolytic abilities in the absence of Bach2. The enhanced cytotoxicity of effector CTLs lacking Bach2 is caused by differences in the initial differentiation state and enlarged cytolytic granules [17].

According to published findings, Bach2-deficient T cells have robust effector capabilities, but downregulating Bach2 expression can impair their movement ability. The relevance of Bach2 in these processes must be validated by additional research because the majority of T-cell trafficking and functional activities fall outside the scope of cytokine production in health or disease.

##### Bach2-mediated regulation of memory CD8 + T cells

Compared with initiating naive T-cell precursors, memory CD8 + T cells have several distinct features. Memory CD8 + T lymphocytes have a considerably greater frequency of persistence in the blood than do other T lymphocytes, and they typically do not require additional differentiation to activate immediate effector activities [18].

Bach2 was found to be a transcription factor whose expression changes with age in lymphocyte subsets, as was PRDM1, the key epigenetic gene involved in terminal T-cell differentiation [12]. As a transcriptional regulator generated from B cell and T cell differentiation, PRDM1 is critical for T cell-mediated immunosuppression. By interacting with other transcription factors, PRDM1 controls the expression of multiple interleukins in effector T cells. The expression of PRDM1 also has substantial effects on the prognosis of various types of cancers [19]. In this study, Bach2 promoted both the development and recall of proliferating memory CD8 + T cells through PRDM1, suggesting that Bach2 and PRDM1 are involved in immune senescence [20]. During the GWAS analysis of CD8 + T cells, a perturbation network was constructed that included Bach2, which is upstream of the regulatory structure. Studies have confirmed that overexpression of Bach2 significantly promotes the proliferation of memory CD8 + T cells both in vitro and in vivo. In contrast, knockdown of Bach2 inhibited the recall proliferation of transduced memory T cells. Additionally, detailed analysis of the Bach2-BLIMP1 pathway provides new insights into memory CD8 + T-cell development. Thus, as a key regulatory transcription factor, Bach2 also promotes the proliferation of memory CD8 + T cells [20]. In addition, Bach2 expression was upregulated in the early phases of memory T-cell formation, indicating that Bach2 may contribute to establishing immune memory induced by vaccines [21].

Studies have shown the importance of killer lectin-like receptor G1 (KLRG1) + effector CD8 + T cells in promoting multifunctional memory cells and long-term protective immunity. These authors also suggested that the developmental plasticity of KLRG1 + effector CD8 + T cells is important for promoting functionally versatile memory cells and long-term protective immunity. As a transcriptional repressor, Bach2 not only indirectly regulates the development of memory CD8 + T cells but also regulates the developmental plasticity of KLRG1 + effector cells [22]. There is strong evidence that Bach2 stimulates the induction of memory T cell precursors, which in turn leads to the development of memory CD8 + T cells; this is because Bach2 expression in effector T cells results in a phenotype characterized by low KLRG expression [20], which is believed to be a precursor of central memory T cells with high proliferative potential [23]. When CD8 + T cells express KLRG1 during the process of differentiation into effector cells, they begin to deteriorate and die. The biological and prognostic significance of KLRG1-defined CD8 T cell subsets in follicular lymphoma (FL) has been determined, which implies that managing CD8 + T-cell differentiation in FL may improve the clinical prognosis of lymphoma patients by enhancing the antitumour immune response [24].

In conclusion, Bach2 influences immunological senescence and has the potential to be useful in clinical targeted therapy by promoting the proliferation of CD8 + T memory cells through PRDM1, KLRG1, and other factors, which impact the process of immune senescence.

##### Bach2-mediated regulation of CD8 + T cell exhaustion

The exhaustion state of CD8 + T cells was subsequently explored as we continued to investigate the role of Bach2 in the management of CD8 + T cells. The generation of memory CD8 + T cells is inhibited, and the effector capabilities of CD8 + T cells deteriorate when antigens endure chronic viral infection or malignancy. Exhaustion is the term used to describe this state of CD8 + T cells. Progressive and hierarchical effector function impairment, prolonged expression of inhibitory receptors, alterations in the epigenetic and transcriptional landscape, and metabolic reprogramming are the hallmarks of CD8 + T-cell exhaustion [17]. Yao et al. demonstrated that Bach2 is required for the differentiation of stem-like CD8 + T cells during chronic viral infection. Bach2 may affect CD8 + T cell differentiation through the RUNX3 pathway and PRDM1 expression. Bach2 inhibited the molecular program that causes terminal exhaustion by silencing the epigenome and repressing transcription. They also demonstrated that the transcriptional repressor Bach2 establishes the epigenetic and transcriptional landscape of stem-like CD8 + T cells after chronic viral infection [25]. The Bach2-mediated downregulation of transcriptional signalling in stem cells is consistent with the discovery that Bach2 decreases the expression of coinhibitory receptors, such as programmed cell death protein 1 (PD-1), T cell-containing immunoglobulin and mucin domain-3 (TIM-3) and T cell immunoreceptor with Ig and ITIM domains (TIGIT) [26]. Blockade of the PD-1 pathway has emerged as a pivotal strategy for reinvigorating exhausted CD8 + T cells, thereby enhancing the body’s ability to exert better control over infections and cancers [27]. Additionally, Tim-3 has been identified as a marker of T-cell exhaustion in both cancer and chronic infections, especially when it is coexpressed with PD-1, indicating a synergistic effect on T-cell impairment [28]. Further research revealed that TIGIT, another inhibitory receptor, was positively correlated with PD-1 and Tim-3 on CD8 + T cells [29]. The finding that overexpression of Bach2 downregulated the above immune checkpoint molecules in stem-like CD8 + cells further supported the critical role of Bach2 in preventing CD8 + T-cell terminal exhaustion. In terms of the mechanism of exhaustion, overexpression of Bach2 inhibits the function of IRF4 and AP-1 family transcription factors, which promote amplification, effector differentiation and exhaustion [30].

### Related diseases

There are numerous pathways and mediators that lead to the various presentations of immune diseases, including Bach2, which is a multiple sclerosis susceptibility gene. To further confirm the critical role of Bach2 in the immune system, genome-wide association studies combined with meta-analyses of genome-wide analysis have been conducted, and the results indicate that Bach2 is associated with numerous immune-related diseases [6]. It is particularly important for the development and treatment of haematological illnesses such as chronic lymphocytic leukaemia, tumours, infectious diseases, and autoimmune diseases.

Studies have shown that Bach2 is closely related to immune senescence. Research comparing age-related alterations in lymphocytes from healthy blood donors and patients with chronic lymphocytic leukaemia (CLL) revealed that Bach2 gene expression was downregulated with age. Moreover, CD8 + T cells from untreated CLL patients showed a further decrease in Bach2 mRNA expression. Additionally, Bach2 gene expression and CD8 + T cell quantity and activity were strongly correlated in the present study. Bach2 mRNA expression was also significantly decreased in CD4 + T cells, which likely affects normal functions [31]. Immune system dysfunction, which is mostly characterized by deficient humoral and cellular immunity, is a critical fundamental aspect of the pathophysiology of CLL. B-cell lymphoma-2 (BCL-2) expression in the T cells of CLL patients is thought to be associated with immunosuppression, according to single-cell RNA sequencing (scRNA-seq) data, which revealed that Bcl2 + CD8 + T cells exhibit the genetic traits of exhausted CTLs. In patients with chronic lymphocytic leukaemia, Bcl2 also encourages cell exhaustion and inhibits CD8 + T cell killing activity via the increase in Treg abundance [32]. The results revealed that Bach2 knockdown enhances resistance to age-related apoptosis in normal cells. These findings provide solid foundations for further investigations into the mechanism of immunosenescence by highlighting the potential involvement of Bach2 in the immunosenescence process [31]. We can acquire more in-depth knowledge and insight into the potential therapeutic mechanisms of Bach2 and other transcriptional regulators in the pathophysiology of UC by evaluating the regulatory pathways of Bach2 and other DEGs in UC [33].

Bach2 is also a crucial factor for promoting immune suppression within tumours. Through a mechanism known as Treg-mediated inhibition of intratumoral CD8 + T cells and interferon γ (IFN-γ), Bach2 supports tumour immunosuppression. As previously demonstrated, Bach2 inhibits the helper cell transcriptional pathway to promote Foxp3 + Treg development. Thus, the Foxp3 + Treg population exhibited a trend toward a significant reduction in tumour density in Bach2-deficient animals reveals therapeutic targets for reversing cancer immunosuppression and is unquestionably a critical part of the molecular pathway involved in tumour immunosuppression. There is evidence that Bach2 suppresses the induction of appropriate innate and adaptive immunity and promotes tumour growth in a tumour cell-extrinsic manner. As a result, Bach2 restricts the growth of CD8 + effector T cells and the expression of their effector cytokines in malignancies [34]. Thus, bach2 can be explored as a potential therapeutic target for cancers. It has been discovered that Bach2 is required for immunosuppression in cancers. It can successfully suppress the ability of CD8 + T lymphocytes to defend against tumours. The molecular pathways underlying tumour immunosuppression are recognized by these results, and they also present targets for immune-based cancer therapies that attempt to alter harmful immunosuppression [34].

Additionally, Bach2 is crucial for the immunological prevention of vaccination. When IL-7 was added to the traditional tuberculosis vaccine, treatment with the recombinant adeno-associated virus (rAAV)-IL-7 mixed subunit vaccine resulted in significantly increased Bach2 expression at the beginning of CD8 + T-cell development. Moreover, the TB subunit vaccine encourages the development of long-lasting memory T cells and long-term antibodies through the combined action of IL-7 and Bach2 on CD4 + and CD8 + T cells [21]. The principal organ involved in Mycobacterium tuberculosis infection is the lung, where it primarily targets antigen-presenting cells such as macrophages and dendritic cells before progressing to the granulomatous infection stage. The development of tuberculosis vaccines has centred on activating cells that handle various immune reactions. Vaccines that can trigger atypical T-cell responses in addition to regular CD4 + and CD8 + T cell responses have become the most recent frontier in the fight against tuberculosis [35].

The differentially expressed genes (DEGs) were significantly enriched in target genes such as Bach2 according to the ulcerative colitis gene expression data, and signal transduction was markedly improved. The oestrogen receptor (ESR) and nongenomic oestrogen signalling pathways are both mediated by the Bach2 target gene. They also made the further assumption that the deregulation of these signals would subsequently cause colorectal cancer. Furthermore, the accumulation of CD8 + cells may be connected to the acquired immunological response in ulcerative colitis (UC), which is in line with the previously described connection between Bach2 and CD8 + T cells [33]. Repeated antigen exposure can trigger a rapid immune response in lymphocytes that have previously encountered colonic antigens, such as tissue-resident memory CD8 + T cells. The cellular phenotypes of these cells as well as the mechanisms through which they govern inflammation and the immune system have been defined. These findings imply that although UC-associated CD8 + effector T cells can harm tissue and generate tumour necrosis factor, the signals acquired by effector cells may inhibit their ability to manage inflammation. Correspondingly, CD8 + cells were shown to indicate long-term stimulation in UC patients. This finding may point to a molecular mechanism for sustained T-cell responses under chronic, long-lasting activation in which canonical pathways could facilitate the process through which these cells preserve their defence functions even in the absence of antigen-specific stimulation. Future research must focus on the precise processes through which gene polymorphisms are connected to inflammatory bowel disease (IBD) and on how to distinguish phenotypic abnormalities among monoclonal clones [36].

In summary, Bach2 is strongly linked to the pathophysiology and development of various immunological disorders, infectious illnesses, and malignancies (Table 1). Thus, whether Bach2 can be a clinically valuable therapeutic target warrants further investigation in basic and clinical research.

**Table 1:** Summary of Bach2 modulation of CD8 + T cells in immune diseases

Immune diseases	Related area	Funtion of Bach2	Source/Methods	Ref
Chronic lymphocytic leukemia	Immune senescence	Bach2 downregulate with aging and reduced cytotoxic activity of CD8 + T cells	Human lymphocyte subsets	[31]
Cancer	Immunosuppression	Bach2 restricts the development of CD8 + effector T cells	Bach2-deficient mice	[34]
Tuberculosis	Vaccine and immune protection	The combination of Bach2 and IL-7 by the vaccine promoted CD8 + T cell development	C57BL/6 mice	[21]
Ulcerative colitis	Immune responses	Bach2-mediated differentially expressed genes and CD8 + T cell aggregation are associated with acquired immune responses	DEGs and DETFs from GEO database	[33]
	Sustained T cell responses	Long-term stimulation is a feature of CD8 + effector T cells	Single-cell transcriptomics of human colonic CD8+ T cells	[36]

## Conclusions and perspectives

As an important line of defence in the human immune system, specific CD8 + T cells can recognize and eliminate virus-infected cells, which is highly important for resisting malignant tumours, immune disorders, viral infections and other diseases. Bach2 is an essential factor in the transcriptional and epigenetic programs of stem-like CD8 + T cells during sustained viral infection [25]. To effectively control viral infection, Bach2 regulates the transcriptional response of CD8 + T cells to TCR signals [7]. Overall, we are primarily committed to systematically outlining the crucial regulatory role of Bach2 in the development and function of CD8 + T lymphocytes.

Despite outlining the various activities of Bach2 in the differentiation of CD8 + T cells, we also focused on the participation of Bach2 in the differentiation of CD8 + T cells in the form of disorders associated with terminal exhaustion and exhaustion. Finally, identifying the vital regulatory function of Bach2 in CD8 + T cells could assist in identifying metabolic targets for developing and treating immunological and malignant cancers. Since Bach2 is known to modulate the differentiation and activation of CD4 + T cells, further comprehensive consideration of the impact of Bach2 on different T cell subsets may contribute to better evaluating its potential clinical applications.

## Data Availability

No datasets were generated or analysed during the current study.

## Abbreviations

TFs: Transcriptional factors
CSR: Class switching recombination
SHM: Somatic hypermutation
Blimp-1: B lymphocyte-induced maturation protein 1
PRDM1: PR domain zinc finger protein 1
MARE: Maf recognition element
AP-1: Activator protein-1
TCR: T cell receptor
IRF4: Interferon regulatory factor 4
Tregs: Regulatory T cells
Th2: T helper 2 cell
IL-10: Interleukin 10
CCR4: C-C Motif Chemokine Receptor 4
ST-2: Growth stimulation expressed gene 2
S100a: S100 calcium binding protein a
CTL: Cytotoxic T lymphocyte
IL-7: Interleukin 7
KLRG1: Killer lectin-like receptor G1
PD-1: Programmed cell death protein 1
TIM-3: T cell-containing immunoglobulin and mucin domain-3
TIGIT: T cell immunoreceptor with Ig and ITIM domains
Tex^stem^: Stem cell-like exhausted T cells
CLL: Chronic lymphocytic leukemia
BCL-2: B-cell lymphoma-2
IFN-γ: Interferonγ
rAAV: Recombination adeno-associated virus
DEGs: Differentially expressed genes
ESR: Estrogen receptor
UC: Ulcerative colitis
IBD: Inflammation bowel disease
